# Magnetic skyrmion transistor: skyrmion motion in a voltage-gated nanotrack

**DOI:** 10.1038/srep11369

**Published:** 2015-06-18

**Authors:** Xichao Zhang, Yan Zhou, Motohiko Ezawa, G. P. Zhao, Weisheng Zhao

**Affiliations:** 1 Department of Physics, University of Hong Kong, Hong Kong, China; 2 Center of Theoretical and Computational Physics, University of Hong Kong, Hong Kong, China; 3 Department of Applied Physics, University of Tokyo, Hongo 7-3-1, Tokyo 113-8656, Japan; 4 College of Physics and Electronic Engineering, Sichuan Normal University, Chengdu 610068, China; 5 Key Laboratory of Magnetic Materials and Devices, Ningbo Institute of Material Technology & Engineering, Chinese Academy of Sciences, Ningbo 315201, China; 6 Fert Beijing Institute, Beihang University, Beijing, China

## Abstract

Magnetic skyrmions are localized and topologically protected spin configurations, which are of both fundamental and applied interests for future electronics. In this work, we propose a voltage-gated skyrmion transistor within the well-established framework of micromagnetics. Its operating conditions and processes have been theoretically investigated and demonstrated, in which the gate voltage can be used to switch on/off a circuit. Our results provide the first time guidelines for practical realization of hybrid skyrmionic-electronic devices.

Magnetic skyrmions are particle-like topological solitons, which have been experimentally observed in both non-centrosymmetric bulk ferromagnets and magnetic thin film with asymmetric interfaces in proximity of heavy non-magnetic metal with strong spin-orbit interaction^1^,^2^,^3^,^4^,^5^,^6^,^7^,^8^,^9^,^10^,^11^,^12^. Since the experimental observations of magnetic skyrmion in 2009^7^, this topologically stable nanomagnetic object has received a growing interest in the fields of nanomagnetism and spintronics. Magnetic skyrmions can be used as information carrier in the next-generation information processing and data storage devices due to its remarkable stability, extremely compact size and very low energy-cost in moving them in nanostructures^5^,^6^,^13^,^14^,^15^,^16^,^17^,^18^,^19^,^20^,^21^,^22^. Skyrmionics refers to the emerging technologies based on magnetic skyrmion as information carrier in the interdisciplinary fields of spintronics and nanoelectronics.

To realize and eventually commercialize skyrmionics for future electronics, various challenges need to be solved such as creating and annihilation of skyrmions^6^,^17^,^19^,^23^, conversion of skyrmions with different helicity and vorticity^15^,^20^, efficient transmission and read-out of skyrmions^6^,^16^,^17^,^18^,^22^, *etc*. In this paper, we explicitly address the critical problem of voltage control of magnetic skyrmion, in which the perpendicular magnetic anisotropy (PMA) in the gate region is locally controlled by an applied electric field due to the charge accumulations^24^,^25^. We study the operating conditions of such skyrmion transistors by varying the material parameters such as the PMA and interface-induced Dzyaloshinskii-Moriya interaction (DMI)^26^,^27^. This study enables the design and integration of magnetic skyrmions in conventional Complementary Metal-Oxide-Semiconductor (CMOS) circuitry and will trigger more experimental investigations in this research direction.

## Results

**Figure 1:**
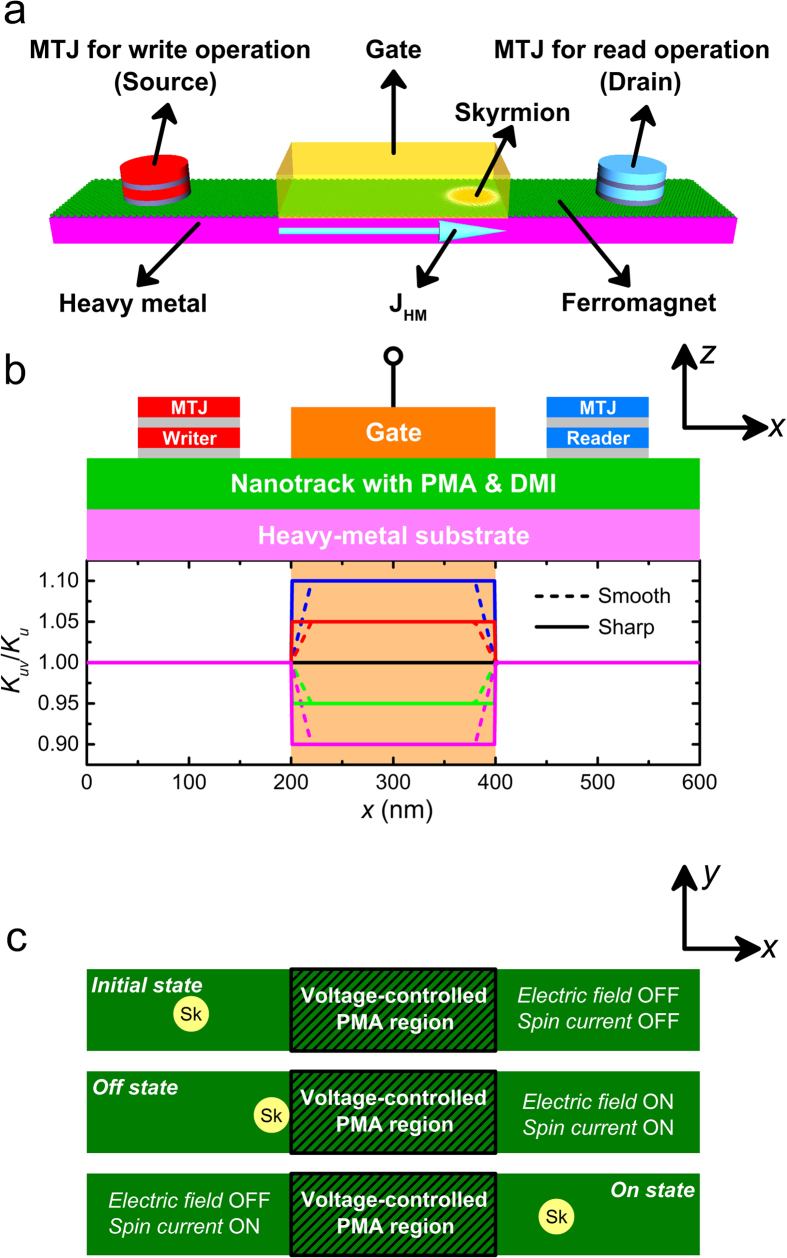
(**a**) Design of the skyrmion transistor. The skyrmion is firstly created by the MTJ writer placed at the left side (*i.e.* the source) and then moves towards the right driven by the spin current, which can be detected when it reaches below the MTJ reader placed at the right side (*i.e.* the drain). The charge current J_HM_ flows through the heavy metal along the x-direction, which leads to the generation of spin current perpendicularly injected to the ferromagnet due to the spin Hall effect. (**b**) Schematic view (*xz*-plane) of the spin-polarized current-driven prototype of the skyrmion transistor. The blue rectangle indicates a nanotrack (length = 600 nm, width = 100 nm and thickness = 1 nm) with perpendicular magnetic anisotropy (PMA) of *K*_*u*_ = 0.8 MJ/m^3^ and the interface-induced Dzyaloshinskii-Moriya interaction (DMI) of *D* = 3.5 mJ/m^2^. The orange rectangle indicates the top electrode for locally changing PMA. The graph presents the variation profile of the PMA of the nanotrack where the PMA of the voltage-gated region *K*_*uv*_ can be tuned. Both smooth and sharp transitions are illustrated in the schematics. (**c**) Schematic view (*xy*-plane) of three states of the skyrmion transistor: *initial*, *off* and *on*. *Initial* state: both the electric field and spin current are turned off; the skyrmion keeps its position on the left side of the nanotrack. *Off* state: both the electric field and spin current are turned on. The spin current drives the skyrmion moving towards the right, while the electric field, which results in the change of PMA in the voltage-gated region, leads to the termination of the skyrmion when it approaches the voltage-gated region. *On* state: the electric field is turned off but the spin current is turned on. The skyrmion driven by the spin current passes the voltage-gated region and reaches the right side of the nanotrack.

With the configuration of applying a gate voltage in the center region of a magnetic nanotrack, the prototype of spin-polarized current-driven skyrmion transistor have been investigated within the well-established framework of micromagnetics. Figure 1 shows the schematic of the voltage-gated nanotrack (600 × 100 × 1 nm^3^) for the spin current-driven prototype of the transistor (see Methods). The skyrmion is initially created by the magnetic tunnel junction (MTJ) writer placed at the left side of the nanotrack (*x* = 90 nm) and then moves towards the right side driven by the spin current, which will be detected by the MTJ reader placed at the right side due to the induced change of tunneling magnetoresistance (TMR)^22^. The spin current *j* of 5–10 MA/cm^2^ is injected from the heavy-metal substrate with spin polarization oriented along *y* direction, due to the spin Hall effect (SHE)^17^,^18^. Within the region of 200 nm < *x* < 400 nm, the PMA value *K*_*uv*_ is controlled by locally applied electric field *E* based on a linearly changing relationship^24^,^28^,^29^, *i.e.*, *K*_*uv*_ = *K*_*u*_ + Δ*K*_*uv*_*E*, which varies between 0.9 *K*_*u*_ and 1.1 *K*_*u*_ (see Methods for all parameters used in this work).

As shown in Fig. 1c, we focus on two non-trivial working states of the voltage-gated skyrmion transistor: *off* and *on*. *Off* state: both the electric field and the spin current are turned on; the spin current drives the skyrmion moving towards the right, while the electric field changes the PMA in the voltage-gated region (*K*_*uv*_ ≠ *K*_*u*_) and creates the energy barrier between the left and right sides of the nanotrack, leading to the termination of the skyrmion when it approaches the voltage-gated region. *On* state: the spin current is turned on but the electric field is turned off (*K*_*uv*_ = *K*_*u*_). The skyrmion driven by the spin current passes the voltage-gated region and reaches the right side of the nanotrack.

**Figure 2:**
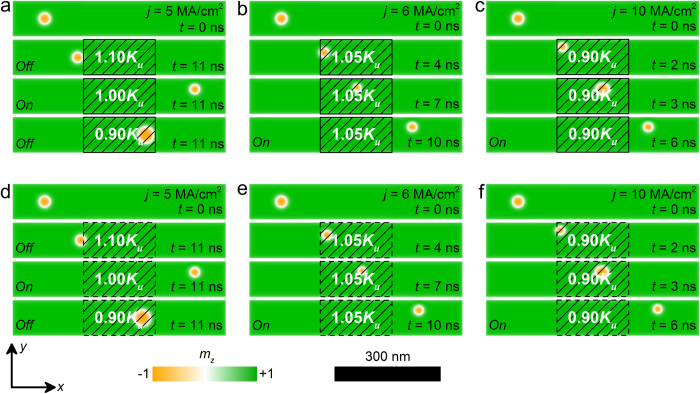
The top-view of the nanotracks under different spin current density *j* as well as different voltage-controlled PMAs K_*uv*_ in the voltage-gated region with sharp transition profile at selected times : (**a**) *j* = 5 MA/cm^2^, *K*_*uv*_ = 1.1 *K*_*u*_, 1.0 *K*_*u*_ or 0.9 *K*_*u*_; (**b**) *j* = 5 MA/cm^2^, *K*_*uv*_ = 1.05 *K*_*u*_; (**c**) *j* = 10 MA/cm^2^, *K*_*uv*_ = 0.9 *K*_*u*_. The top-view of the nanotracks under different *j* and *K*_*uv*_ in the voltage-gated region with smooth transition profile at selected times: (**d**) *j* = 5 MA/cm^2^, *K*_*uv*_ = 1.1 *K*_*u*_, 1.0 *K*_*u*_ or 0.9 *K*_*u*_; (**e**) *j* = 5 MA/cm^2^, *K*_*uv*_ = 1.05 *K*_*u*_; (**f**) *j* = 10 MA/cm^2^, *K*_*uv*_ = 0.9 *K*_*u*_. The colour scale denotes the out-of-plane component of the magnetization. The black-line shadows represent the voltage-controlled PMA region.

Figure 2 shows the top-view of the nanotrack of the skyrmion transistor under different spin current densities as well as different voltage-controlled PMA *K*_*uv*_ in the voltage-gated region. Here, we considered both the sharp (Fig. 2a–c) and smooth (Fig. 2d–f) transitions of PMA at the borders of the voltage-gated region, as shown in Fig. 1a (also see Methods). For both cases as shown in Fig. 2a,d, the skyrmion moves from the left side to the right side of the nanotrack within 11 ns when *j* = 5 MA/cm^2^ and *K*_*uv*_ = 1.00 *K*_*u*_. While when  *K*_*uv*_ = 1.10 *K*_*u*_, the skyrmion is pinned when it meets the first border of the voltage-gated region. When *K*_*uv*_ = 0.90 *K*_*u*_, the skyrmion passes the first border, but it is pinned at the second border of the voltage-gated region. However, when *j* is larger than a certain threshold value, the skyrmion can pass the voltage-gated region even when *K*_*uv*_ ≠ *K*_*u*_, as shown in Fig. 2b,c,e,f. We have further checked that the simulation results are not sensitive to either sharp or smooth transition profiles of PMA. Hence, in this work we focus on the results based on the sharp transition profile of PMA for simplicity.

**Figure 3:**
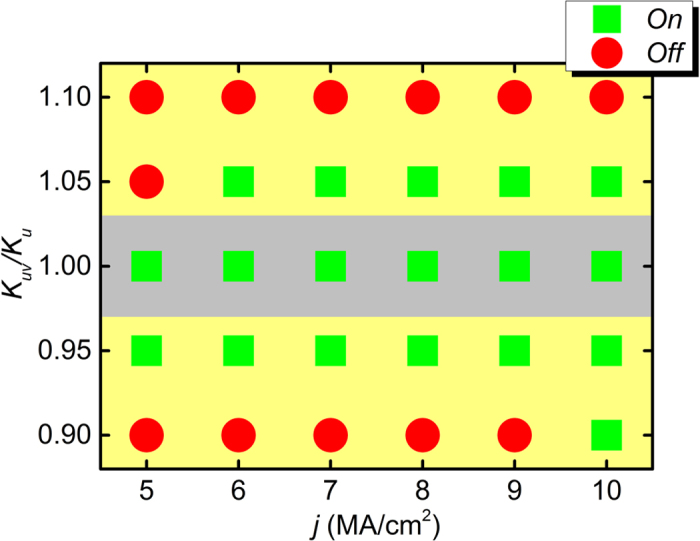
Working window of the spin-polarized current-driven voltage-gated skyrmion transistor. The green square denotes the *On* state, *i.e.*, the skyrmion passes the voltage-gated region moving from the left side to the right side of the nanotrack. The red circle denotes the *Off* state, *i.e.*, the skyrmion cannot pass the voltage-gated region and stops at the rest of the right side of the nanotrack.

Figure 3 shows the working window of the voltage-gated skyrmion transistor at different electric fields and spin currents. Obviously, when the electric field is turned off, *i.e.*, *K*_*uv*_ = 1.00 *K*_*u*_, the transistor is always in the *On* state, in which the skyrmion driven by the spin current can reach the right side of the nanotrack ultimately. When the electric field is turned on and *K*_*uv*_ is increased to 1.05 *K*_*u*_, the transistor state is *Off* when *j* = 5 MA/cm^2^, while when *j* = 6–10 MA/cm^2^, the transistor is in the state of *On*. As *K*_*uv*_ further increases to 1.10 *K*_*u*_, the transistor is always in the state of *off*, even when *j* = 10 MA/cm^2^. Moreover, when the electric field is turned on and *K*_*uv*_ decreases to 0.95 *K*_*u*_, the transistor remains the state of *On* for *j* = 5–10 MA/cm^2^. When *K*_*uv*_ is reduced to 0.90 *K*_*u*_, the state of the transistor switches from *Off* to *On* when *j* is increased to 10 MA/cm^2^. Hence, we can see if *K*_*uv*_ ≠ *K*_*u*_, the skyrmion may be pinned by the barrier induced by different PMA in the nanotrack, resulting in the *Off* state of the transistor. However, a driving current larger than certain threshold can prevent the skyrmion from pinning, resulting in the state of *On*, for instance, when *K*_*uv*_ = 1.05 *K*_*u*_, when *j* ≥ 6 MA/cm^2^, the skyrmion can pass through the voltage-gated region without stopping.

**Figure 4:**
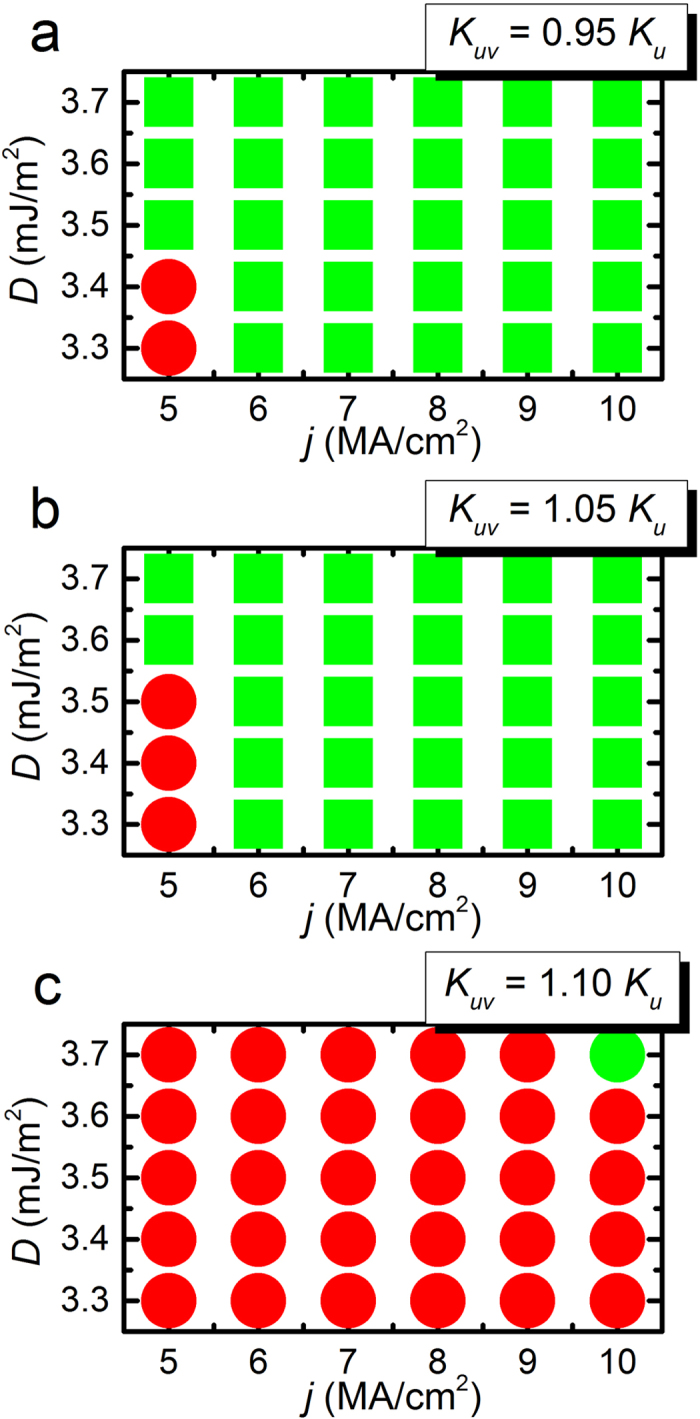
(**a**) Working window of the spin-polarized current-driven voltage-gated skyrmion transistor at different interface-induced Dzyaloshinskii-Moriya interactions (DMI) and spin current densities with fixed voltage-controlled PMA *K*_*uv*_ = 0.95 *K*_*u*_ in the voltage-gated region. (**b**) Working window of the skyrmion transistor at different DMI and spin current densities with fixed voltage-controlled PMA *K*_*uv*_ = 1.05 *K*_*u*_. (**c**) Working window of the skyrmion transistor at different DMI and spin current densities with fixed voltage-controlled PMA *K*_*uv*_ = 1.10 *K*_*u*_. The green square denotes the *On* state, *i.e.*, the skyrmion passes the voltage-gated region moving from the left side to the right side of the nanotrack. The red circle denotes the *Off* state, *i.e.*, the skyrmion cannot pass the voltage-gated region and stops at the rest of the right side of the nanotrack.

Figure 4 shows the working windows of the voltage-gated skyrmion transistor at different DMI strengths and spin currents with fixed electric field. It can be seen from Fig. 4a, when *K*_*uv*_ = 0.95 *K*_*u*_, the transistor is in the state of *Off* for *D* = 3.3–3.4 mJ/m^2^ and *j* = 5 MA/cm^2^. When *D* > 3.4 mJ/m^2^ or *j* > 5 MA/cm^2^, the transistor is working in the state of *On*. Similarly, as shown in Fig. 4b, if *K*_*uv*_ = 1.05 *K*_*u*_, the transistor is in the state of *On* when *D* > 3.5 mJ/m^2^ or *j* > 5 MA/cm^2^, and in the state of *Off* when *D* = 3.3–3.5 mJ/m^2^ and *j* = 5 MA/cm^2^. Figure 4c shows that, when *K*_*uv*_ = 1.10 *K*_*u*_, the transistor is in the state of *On* for *D* = 3.7 mJ/m^2^ and *j* = 10 MA/cm^2^, while when *D* < 3.7 mJ/m^2^ or *j* < 10 MA/cm^2^, the transistor has a stable *Off* state.

## Discussion

The energy and the radius of the skyrmion strongly depend on the magnetic anisotropy. The energy is given by


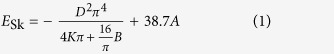


and the radius is given by


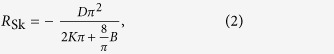


where *D* is the magnitude of the DMI, *A* is the exchange constant, *B* is the magnetic field and *K* is the PMA constant. If the magnetic anisotropy increases, the energy of the skyrmion increases and the radius of the skyrmion decreases. On the other hand, if the magnetic anisotropy is reduced, the energy of the skyrmion decreases and the radius of the skyrmion increases. The skyrmion dynamics is well understood in terms of the Thiele equation


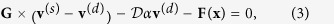


where 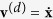 is the drift velocity and **v**^(*s*)^ is the velocity induced by the spin current. **G** = (0, 0, *G*) is the gyromagnetic coupling vector representing the Magnus force. The Magnus force is proportional to the Pontryagin number, *G* = 4π*Q*, with the Pontryagin number being


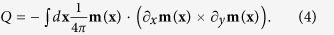


∇ represents the dissipative force. *V*(**r**) is the confining potential induced by the sample edges. *α* is the Gilbert damping constant. **F**(**x**) = ∇*V*(**x**) is the force induced by potential. The potential is given by the local energy of a skyrmion. Hence, it is easy to understand that if *K*_*uv*_ = *K*_*u*_, no potential barrier exists in the nanotrack, the skyrmion driven by spin current moves from the left to the right side of the nanotrack smoothly (see Fig. 2a and Supplementary Movie 1). If *K*_*uv*_ > *K*_*u*_, the left border of the voltage-gated region acts as a potential barrier, which cannot be overcome by a skyrmion without enough driving force from the spin current (see Fig. 2a and Supplementary Movie 2). If *K*_*uv*_ < *K*_*u*_, the left border of the voltage-gated region acts as a potential well and the right boundary acts as a potential barrier. Therefore, in this case, the skyrmion can pass the potential well but cannot pass the potential barrier (see Fig. 2a and Supplementary Movie 3).

However, when *j* is larger than a certain threshold, the driving force on the skyrmion is strong enough to make the skyrmion overcome the potential barrier, as shown in Fig. 2b,c (see Supplementary Movies 4 and 5). It should be noted that the skyrmion shrinks in the voltage-gated region with *K*_*uv*_ > 1.00 *K*_*u*_ and expands in the voltage-gated region with *K*_*uv*_ < 1.00 *K*_*u*_, which is consistent with the above picture.

**Figure 5:**
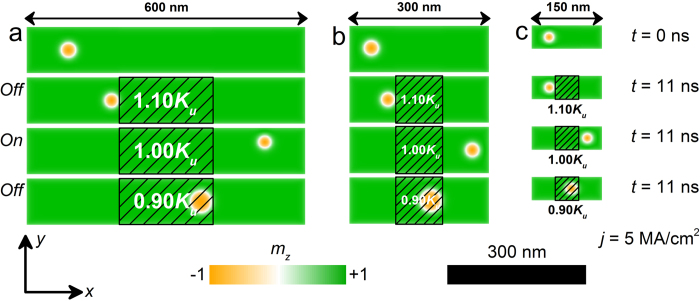
The top-view of the nanotracks with different sizes under the same working conditions at selected times. The sizes of the nanotracks are: (**a**) 600 × 100 × 1 nm^3^; (**b**) 300 × 100 × 1 nm^3^; (**c**) 150 × 50 × 1 nm^3^. The colour scale denotes the out-of-plane component of the magnetization. The black-line shadows represent the voltage-controlled PMA region.

In addition, as shown in Fig. 5, we have investigated that the size effect of the skyrmionic transistor. It can be seen from Fig. 5b, where the size of the skyrmionic transistor is decreased from 600 × 100 × 1 nm^3^ to 300 × 100 × 1 nm^3^, the skyrmion moves from the left side to the right side of the nanotrack within 11 ns when *j* = 5 MA/cm^2^ and *K*_*uv*_ = 1.00 *K*_*u*_. While when *K*_*uv*_ = 1.10 *K*_*u*_, the skyrmion is pinned when it meets the first border of the voltage-gated region. When *K*_*uv*_ = 0.90 *K*_*u*_, the skyrmion passes the first border, but it is pinned at the second border of the voltage-gated region. Fig. 5c shows the results when the size of the skyrmionic transistor further reduces to 150 × 50 × 1 nm^3^ under the same working conditions, which matches well with that of the demonstration model shown in Fig. 5a, indicating the good scalability of the skyrmionic transistor model.

In conclusion, we have investigated the skyrmionic transistor in the framework of micromagnetics. We have shown that whether a skyrmion passes or not can be controlled by the gate voltage through the modulation of PMA in the gate region. We have explicitly determined the parameter space where a skyrmion passes as a function of the injected current and the PMA. This skyrmion transistor could be used as an important component in the hybrid skyrmionic-electronic devices.

## Methods

The three-dimensional micromagnetic simulations are performed by using the Object Oriented MicroMagnetic Framework (OOMMF)^30^ with extended Dzyaloshinskii-Moriya interaction (DMI) module^31^. The time-dependent magnetization dynamics is determined by the well-known Landau-Lifshitz-Gilbert (LLG) equation including spin transfer torque^20^,^25^,^30^,^32^,^33^,^34^,^35^,^36^,^37^. The average energy density contains the exchange energy, the anisotropy energy, the applied field (Zeeman) energy, the magnetostatic (demagnetization) energy and the DMI energy terms.

In all simulations, the thickness of the magnetic nanotracks is 1 nm. The length of the nanotracks is varied from 150 nm to 600 nm, while the width is varied between 50 nm and 150 nm. Typical magnetic parameters adopted from Refs.17 and 18 are used in the simulation: saturation magnetization *M*_*S*_ = 580 kA/m, exchange stiffness *A* = 15 pJ/m, interface-induced DMI^26^,^27^ constant *D* = 3.5 mJ/m^2^, perpendicular magnetic anisotropy (PMA) constant *K*_*u*_ = 0.8 MJ/m^3^ and gyromagnetic ratio *γ* = −2.211 × 10^5^ m/As unless otherwise specified. The Gilbert damping coefficient *α* is 0.3^17^. All models are discretized into tetragonal cells with the constant cell size of 2 × 2 × 1 nm^3^ in the simulation, which is sufficiently smaller than the domain wall length (4.3 nm) to ensure the numerical accuracy with reasonable computational efficiency. The time step of the simulation is fixed at 0.02 ns.

The initial magnetic states of the nanotracks is relaxed along the +z direction, expect for the tilted magnetization near the edges due to the DMI. At the first stage, a skyrmion is generated at designated spot, *i.e.*, the 20-nm-diametral area under the MTJ writer as shown in Fig. 1a, by the vertical spin-polarized current, and then relaxed to stable/metastable state within a short period of time. It should be noted that the structures of MTJ writer and reader are not modeled in the simulation. For simplicity, we directly simulate the injection of the spin-polarized current pulse with certain current density and polarization rate in the same manner as in Ref. 17. The polarization rate (*P*) of the spin current used in all simulations is 0.4.

## Additional Information

**How to cite this article**: Zhang, X. *et al.* Magnetic skyrmion transistor: skyrmion motion in a voltage-gated nanotrack. *Sci. Rep.* **5**, 11369; doi: 10.1038/srep11369 (2015).

## Supplementary Material

Supplementary Information

Supplementary Movie 1

Supplementary Movie 2

Supplementary Movie 3

Supplementary Movie 4

Supplementary Movie 5
