# Supplementary material for: Magnetic skyrmion transistor: skyrmion motion in a voltage-gated nanotrack
Source: Sci Rep. 2015 Jun 18;5:11369. doi: 10.1038/srep11369 (PMC4471904; doi:10.1038/srep11369)
Supplement: Supplementary Information [file srep11369-s1.pdf]

Magnetic skyrmion transistor: skyrmion motion in a voltage-gated nanotrack

Xichao Zhang[1], Yan Zhou[1, 2 *], Motohiko Ezawa[3], G. P. Zhao[4, 5], W. S. Zhao[6 †]

1. Department of Physics, University of Hong Kong, Hong Kong, China
2. Center of Theoretical and Computational Physics, University of Hong Kong, Hong Kong, China
3. Department of Applied Physics, University of Tokyo, Hongo 7-3-1, Tokyo 113-8656, Japan
4. College of Physics and Electronic Engineering, Sichuan Normal University, Chengdu 610068, China
5. Key Laboratory of Magnetic Materials and Devices, Ningbo Institute of Material Technology & Engineering, Chinese Academy of Sciences, Ningbo 315201, China
6. Fert Beijing Institute, Beihang University, Beijing, China

*E-mail: yanzhou@hku.hk

†E-mail: weisheng.zhao@buaa.edu.cn

SUPPLEMENTARY INFORMATION

Supplementary Movie Captions

In all supplementary movies, the voltage-gated region is denoted by the shadow pattern.

Supplementary Movie 1. The nanotrack with spin current density j = 5 MA/cm^2 as well as voltage-controlled PMA K_{uv} = K_u in the voltage-gated region with sharp transition profile, corresponding to Figure 2a of the main text. The skyrmion passes the gated region.

Supplementary Movie 2. The nanotrack with spin current density j = 5 MA/cm^2 as well as voltage-controlled PMA K_{uv} = 1.1K_u in the voltage-gated region with sharp transition profile, corresponding to Figure 2a of the main text. The skyrmion cannot pass the gated region.

Supplementary Movie 3. The nanotrack with spin current density j = 5 MA/cm^2 as well as voltage-controlled PMA K_{uv} = 0.9K_u in the voltage-gated region with sharp transition profile, corresponding to Figure 2a of the main text. The skyrmion cannot passes the gated region.

Supplementary Movie 4. The nanotrack with spin current density j = 6 MA/cm^2 as well as voltage-controlled PMA K_{uv} = 1.05K_u in the voltage-gated region with sharp transition profile, corresponding to Figure 2b of the main text. The skyrmion passes the gated region.

Supplementary Movie 5. The nanotrack with spin current density j = 10 MA/cm^2 as well as voltage-controlled PMA K_{uv} = 0.9K_u in the voltage-gated region with sharp transition profile, corresponding to Figure 2c of the main text. The skyrmion passes the gated region.
