# Supplementary figures and images for: Magnetic skyrmion transistor: skyrmion motion in a voltage-gated nanotrack
Source: Sci Rep. 2015 Jun 18;5:11369. doi: 10.1038/srep11369 (PMC4471904; doi:10.1038/srep11369)

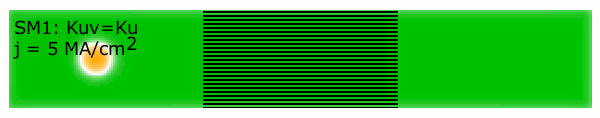

Supplement: Supplementary Movie 1 [file srep11369-s2.gif]

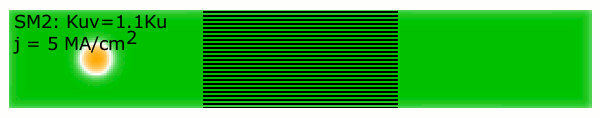

Supplement: Supplementary Movie 2 [file srep11369-s3.gif]

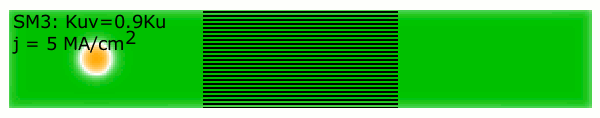

Supplement: Supplementary Movie 3 [file srep11369-s4.gif]

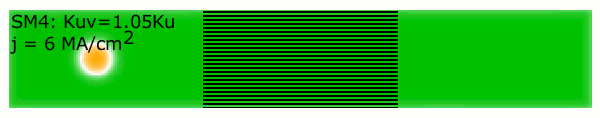

Supplement: Supplementary Movie 4 [file srep11369-s5.gif]

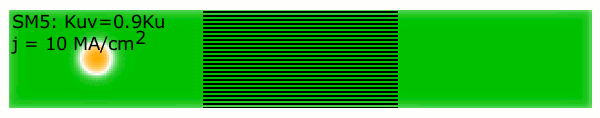

Supplement: Supplementary Movie 5 [file srep11369-s6.gif]
